# Nutrient Sensor mTOR and OGT: Orchestrators of Organelle Homeostasis in Pancreatic *β*-Cells

**DOI:** 10.1155/2020/8872639

**Published:** 2020-12-16

**Authors:** Nicholas Esch, Seokwon Jo, Mackenzie Moore, Emilyn U. Alejandro

**Affiliations:** ^1^Department of Integrative Biology & Physiology, University of Minnesota Medical School, University of Minnesota, Minneapolis, Minnesota, USA; ^2^Department of Surgery, University of Minnesota Medical School, University of Minnesota, Minneapolis, Minnesota, USA

## Abstract

The purpose of this review is to integrate the role of nutrient-sensing pathways into *β*-cell organelle dysfunction prompted by nutrient excess during type 2 diabetes (T2D). T2D encompasses chronic hyperglycemia, hyperlipidemia, and inflammation, which each contribute to *β*-cell failure. These factors can disrupt the function of critical *β*-cell organelles, namely, the ER, mitochondria, lysosomes, and autophagosomes. Dysfunctional organelles cause defects in insulin synthesis and secretion and activate apoptotic pathways if homeostasis is not restored. In this review, we will focus on mTORC1 and OGT, two major anabolic nutrient sensors with important roles in *β*-cell physiology. Though acute stimulation of these sensors frequently improves *β*-cell function and promotes adaptation to cell stress, chronic and sustained activity disturbs organelle homeostasis. mTORC1 and OGT regulate organelle function by influencing the expression and activities of key proteins, enzymes, and transcription factors, as well as by modulating autophagy to influence clearance of defective organelles. In addition, mTORC1 and OGT activity influence islet inflammation during T2D, which can further disrupt organelle and *β*-cell function. Therapies for T2D that fine-tune the activity of these nutrient sensors have yet to be developed, but the important role of mTORC1 and OGT in organelle homeostasis makes them promising targets to improve *β*-cell function and survival.

## 1. Introduction

Analogous to the way in which dysfunctional organs can cause diseases and death, intracellular organelles that deviate from their normal physiological performance can disturb the functions and vitality of the cells in which they reside. As this review will discuss, the pandemic of diabetes in the United States and worldwide is a chief illustrator of this principle. Type 2 diabetes (T2D) is characterized by hyperglycemia [1], hyperlipidemia [2], and key changes in amino acid profiles [3] that are associated with alterations in peripheral insulin sensitivity and *β*-cell function. Important parameters of *β*-cell function include responsiveness to stimulation by nutrients (i.e., glucose and amino acids) and sufficient *β*-cell mass to regulate nutrient homeostasis in the bloodstream. Despite an initial phase of functional adaptation during the development of T2D, *β*-cells gradually lose their ability to regulate homeostasis due to sustained *β*-cell overwork caused by chronic and increasing insulin resistance [4]. Hyperglycemia and hyperlipidemia that accompany these changes are cytotoxic to *β*-cell health and function [5] because they disrupt organelle activities and activate pathways for programmed cell death. Organelle homeostasis is regulated by a vast number of mechanisms, many of which cross-communicate with and regulate one another. In recent years, research has intently focused on uncovering these mechanisms in *β*-cells and learning how the functions of *β*-cell organelles are disrupted by sustained elevations in nutrient levels during prediabetes and T2D. Signals from intracellular nutrient sensors have emerged as particularly important regulators of *β*-cell function during the pathogenesis of T2D [6, 7]. However, the union between organelle dysfunction and nutrient-sensing pathways in *β*-cell failure has not been wholly recognized up to this point. In this review, we summarize how dysregulated signals from anabolic nutrient-sensing pathways during T2D integrate numerous modes of organelle dysfunction within *β*-cells, ultimately leading to their demise. In particular, we review how the activities of the endoplasmic reticulum (ER), mitochondria, lysosomes, and autophagosomes within *β*-cells are influenced by chronic and sustained stimulation of two key nutrient-sensor proteins, the mechanistic target of rapamycin (mTOR) and O-linked N-acetylglucosamine transferase (OGT), and how these changes contribute to systemic *β*-cell failure. The catabolic nutrient sensor AMP-activated protein kinase (AMPK) is also important for *β*-cell function, and AMPK signaling is perturbed during T2D [8, 9]. However, AMPK and organelle homeostasis in *β*-cells is understudied; this is a subject greatly in need of further research. We will address AMPK in this review when relevant, but our discussion of nutrient sensors will largely focus on mTOR and OGT. In addition to these nutrient-sensing proteins, we discuss autophagy and inflammation, two mechanisms that regulate organelle homeostasis and are sensitive to nutrients status. Autophagy acts as an important intracellular process in *β*-cells that is used to maintain healthy pools of organelles, but when this process is disturbed, organelle homeostasis can become disrupted as well. By contrast, inflammation, which is chronically elevated in obesity and T2D, acts as an important extrinsic signal for promoting *β*-cell organelle dysfunction [10].

### 1.1. mTOR Signaling in *β*-Cells

mTOR is one of the primary nutrient-sensitive signaling switchboards that controls *β*-cell metabolism and function. This serine-threonine kinase exists in two distinct complexes within cells: mTOR complex 1 (mTORC1) and mTOR complex 2 (mTORC2). These complexes integrate and coordinate cell responses to signals from growth factors, mitogens, glucose, amino acids, oxygen, intracellular energy levels, and cell stress [11]. mTORC1 is primarily involved in the activation of anabolic pathways, energy utilization, cell growth, and proliferation, whereas mTORC2 largely functions as a regulator of cell survival and cytoskeletal reorganization [11]. Within the *β*-cell specifically, mTORC1 and mTORC2 play critical roles in the regulation of *β*-cell mass, insulin secretion, and maturation [12–20]. Short-term mTORC1 activation is beneficial for insulin biosynthesis and secretion [21] and can improve glucose homeostasis by expanding *β*-cell mass [22]. However, sustained mTORC1 activity in *β*-cells due to genetic manipulation or chronic nutrient excess results in *β*-cell dysfunction and eventual failure [23]. mTORC2 has been studied less extensively than mTORC1 but appears to have critical roles in *β*-cell adaptation to metabolic stress, *β*-cell proliferation, survival, and glucose-stimulated insulin secretion (GSIS) [18, 24]. Crucially, *β*-cells from T2D individuals and mouse models display mTORC1 hyperactivity and mTORC2 hypoactivity [25]. This is in part mediated by chronic hyperglycemia [25] and may also result from elevations in key amino acids during T2D. mTORC1 activity is quite responsive to amino acids and is more sensitive to these nutrients than mTORC2 [26]. T2D, obesity, and insulin resistance have been associated with changes in serum amino acids, specifically elevations in branched-chain and aromatic amino acids [3]. Branched-chain amino acids, especially leucine, are potent activators of mTORC1 and may increase its activity during T2D [27]. In addition to increased nutrient stimulation, the changes in mTOR complex activity may be exacerbated and maintained by negative regulation of mTORC2 by mTORC1. mTORC1 hyperactivity and mTORC2 hypoactivity in the context of T2D have many diverse and complex outcomes on a number of metabolic pathways, but in this review, we will focus on the junctions between mTOR signaling and organelle performance.

### 1.2. The Hexosamine Biosynthetic Pathway and Protein O-GlcNAcylation

In addition to mTOR complexes, OGT is an additional intracellular nutrient sensor of critical importance in the *β*-cell [7]. OGT activity is driven by the hexosamine biosynthetic pathway (HBP), a metabolic pathway that integrates carbohydrate, amino acid, lipid, and nucleotide metabolism. It is estimated that approximately 3-5% of intracellular glucose is shunted into the HBP and that this nutrient is the primary driver of flux, though amino acids and saturated fatty acids also direct the HBP. Metabolic studies have not been performed in *β*-cells to directly quantify the flux of HBP metabolites in response to glucose. However, it is apparent that glucose stimulates HBP in *β*-cells, as metabolites like glucosamine and N-acetylglucosamine (GlcNAc) can recapitulate the effects of glucose on *β*-cell parameters like development [28] and gene expression [29]. In addition to glucose, glucosamine is also an important driver for flux, as this nutrient bypasses the first and rate-limiting enzyme of the HBP, glutamine:fructose-6-phosphate transaminase (GFAT). The ultimate product of the HBP is uridine-diphosphate-N-acetylglucosamine (UDP-GlcNAc). This substrate is used by OGT to glycosylate nuclear, cytoplasmic, and mitochondrial proteins at serine and threonine residues, a process referred to as O-GlcNAcylation. O-GlcNAcylation by OGT is distinct from the complex N- and O-linked glycosylations occurring in the ER and Golgi during protein synthesis [30], and it is used to rapidly and dynamically modify protein activity, stability, and localization in a manner similar to phosphorylation. In fact, O-GlcNAcylation and phosphorylation frequently share protein targets, causing them to work in coordination or competition against each other and produce complex patterns of regulation [31]. Unlike phosphorylation, which is regulated by hundreds of kinases and phosphatases, the job of adding and removing O-GlcNAc is performed by only two enzymes: OGT and O-GlcNAcase (OGA), respectively. The activities of OGT and OGA enzymes are governed by UDP-GlcNAc availability, which depends on the intracellular concentrations of glucose, amino acids, and fatty acids and the activity of HBP enzymes. In addition, multiple forms of cell stress cause changes to global O-GlcNAcylation in an adaptive and cytoprotective manner [32].

*β*-cells demonstrate a marked use of HBP and protein O-GlcNAcylation to fine-tune the coupling of nutrient availability to signaling pathways and organelle activity. OGT is highly expressed in pancreatic islets [33], and perturbations to *β*-cell O-GlcNAcylation through OGT deletion [7, 34] or OGA inhibition [35] cause *β*-cell failure and diabetes. OGA polymorphisms have been linked to T2D as well [36, 37]. Moreover, prolonged hyperglycemia during diabetes causes sustained and excessive O-GlcNAcylation that compromises *β*-cell function [38]. Chronic hyperlipidemia appears to have more complicated effects on islet O-GlcNAcylation, but obesity in humans and high-fat diet (HFD) feeding in rodents appear to diminish the amount of OGT and O-GlcNAcylation in islets [39]. As described in the subsequent sections of this review, dysregulated nutrient sensing through this pathway compromises the activities of several organelles crucial for *β*-cell function.

## 2. Dysfunction of the Endoplasmic Reticulum

The ER functions as a hub of activity for protein translation, modification, and folding, as well as regulation of calcium signaling and homeostasis, among other functions. In *β*-cells, the ER is largely dedicated to oxidative folding of proinsulin before it is trafficked to the Golgi [40]. When the demand for protein folding and modification exceeds the processing capacity of the ER, unfolded proteins accumulate within the ER lumen, and this disruption to ER homeostasis is referred to as “ER stress” [41]. ER stress is also prompted by perturbations to calcium levels within the ER, as many ER-resident proteins responsible for protein folding are calcium-dependent. ER stress is attenuated through a series of negative feedback mechanisms termed “the unfolded protein response” (UPR). The UPR reduces ER stress through (1) transcriptional upregulation of proteins that enhance folding capacity, (2) impairment of translation to reduce protein load within the ER, (3) clearance of excess proteins through ER-associated degradation (ERAD), and, if ER stress is severe, (4) induction of autophagy to selectively target and degrade portions of damaged ER (i.e., ER-phagy) [41]. Additionally, the UPR regulates cell survival and will promote apoptosis when ER stress cannot be mitigated [41]. Though sustained or severe ER stress can be harmful, many secretory cells leverage the UPR induced by mild ER stress to optimize cell function [42]. The ER of *β*-cells in particular functions at the upper limits of its capacity to synthesize insulin, and the UPR induced by postprandial hyperglycemia allows *β*-cells to transiently surpass these limits to increase insulin production and meet the demands of the body during the absorptive state. Moreover, *β*-cells utilize mild ER stress to optimize proliferation [43].

The UPR is directed by three signal-transducing stress sensors located in the ER membrane: inositol-requiring enzyme-1 (IRE1), PKR-like ER kinase (PERK), and activating transcription factor 6 (ATF6) [41]. Activated IRE1 splices an mRNA that encodes a transcription factor for UPR target genes, X-box binding protein-1 (XBP-1) [44] [45]. When activated for sustained periods, IRE1 promotes apoptosis through activation of c-Jun N-terminal protein kinase (JNK) [46]. In *β*-cells, IRE1 also has roles in enhancing insulin biosynthesis in response to acute hyperglycemia [47] and degrading insulin mRNA during prolonged ER stress [48, 49]. PERK stalls global translation by phosphorylating the *α* subunit of eukaryotic translation initiation factor 2 (eIF2*α*), thereby preventing the assembly of ribosome initiation complexes and start codon recognition [50]. Simultaneously, phospho-eIF2*α* promotes selective translation of UPR-encoding mRNAs, including mRNA for activating transcription factor 4 (ATF4) [51]. ATF4 promotes the expression of the proapoptotic transcription factor C/EBP-homologous protein (CHOP), another critical regulator of cell death alongside JNK [52]. Activated ATF6 is cleaved in the Golgi and released into the cytoplasm where it can be trafficked into the nucleus to stimulate transcription of XBP-1, CHOP, and ER protein chaperones [53, 54]. In the *β*-cell, ATF6 also represses insulin transcription [55] and may play a minor role in insulin secretion in response to glucose stimulation [56]. These proteins that facilitate the ER stress response are sensitive to nutrient levels and, as will be discussed in subsequent sections of this review, are finely regulated by nutrient-sensing proteins and pathways in *β*-cells.

During T2D, one avenue by which chronic hyperglycemia and hyperlipidemia mediate *β*-cell failure is excessive ER stress. Culturing primary human islets in high glucose for 48 hours induces the UPR [57], likely due to the increased insulin biosynthesis and chronic stimulation of secretion. Higher insulin production increases the load of nascent proinsulin within the ER, overwhelming the number of ER protein chaperones and inducing the UPR. Furthermore, the necessity for oxidative disulfide bond formation between proinsulin chains promotes the generation of reactive oxygen species (ROS) that can lead to oxidative damage within the ER [40]. Additionally, sustained insulin secretion depletes ER calcium stores [57], further worsening ER function and prolonging ER stress. Though glucotoxicity causes *β*-cell ER stress through multiple mechanisms, lipotoxic conditions are more potent initiators of ER stress [58] with saturated fatty acids being regarded as more potent activators than monounsaturates [58, 59]. Free fatty acids deplete ER calcium and slow calcium uptake from the cytoplasm through the sarcoplasmic/endoplasmic reticulum Ca2^+^-ATPase (SERCA), impairing the function of calcium-dependent ER proteins and leading to an accumulation of misfolded proteins within the ER [58]. Furthermore, high concentrations of palmitate (the major saturated fatty acid in the body) alter membrane fluidity and ER morphology, disrupting ER-to-Golgi trafficking and causing proteins to stall within the ER and stimulate ER stress [60].

### 2.1. *β*-Cell ER Dysfunction in T2D

ER stress and a dysregulated UPR have important roles in the pathophysiology of diabetes. Several genetic contexts predispose individuals to ER stress-induced *β*-cell failure early in life [61, 62], and polymorphisms in ER stress sensors have been linked to T2D in certain populations [63–65]. Importantly, signs of *β*-cell ER stress are evident in *ex vivo* and *in vitro* studies with islets from T2D donors. Immunostaining of islets from T2D patients demonstrate elevations in downstream UPR targets [66], and electron microscopy (EM) imaging reveals significant expansion and morphological changes of the ER of T2D *β*-cells indicative of ER stress [67]. The nuclei of *β*-cells from individuals with T2D and obesity show elevated amounts of the late-stage UPR transcription factor CHOP [68]. *In vitro*, glucotoxic conditions activate the UPR more readily in T2D islets than in islets from healthy donors [67]. Furthermore, transplantation of human islets to euglycemic mice induces mild ER stress and an adaptive UPR in these islets, but transplantation into diabetic mice induces proapoptotic ER stress signaling, suggesting that hyperglycemia may prolong ER stress past the point of beneficial adaptation [69].

Since *in vivo* studies of human islets present significant challenges, rodent models of diabetes have also been useful tools for elucidating the role of ER stress in *β*-cell dysfunction during chronic nutrient excess. Islets from diabetic leptin-receptor-deficient *db/db* mice display signs of ER stress including elevated transcription of key UPR proteins, markers for their downstream targets, and ER morphological changes [66]. *β*-cells from Zucker diabetic fatty (ZDF) rats exhibit increased susceptibility to ER stress-induced by lipotoxic conditions [59]. By contrast, mice with a defective eIF2*α* allele are unable to marshal a sufficient UPR when challenged with a metabolically stressful high-fat diet (HFD), leading to severe glucose intolerance due to poorly regulated ER stress that impairs insulin synthesis [70]. Importantly, the role of ER stress in *β*-cell failure is highlighted by findings that multiple mouse models of T2D can be improved by reductions in *β*-cell CHOP levels, thereby attenuating proapoptotic signals originating from ER stress [7, 71].

### 2.2. mTOR and Endoplasmic Reticulum Dysfunction

The interconnected relationship between mTOR signaling and ER stress has been established [72, 73]. mTORC1 activation can be both an upstream activator of ER stress and a downstream outcome. mTORC1 induces a mild UPR under physiologic conditions by enhancing protein synthesis and therefore increasing the protein load within the ER. Conversely, the ATF6 arm of the UPR activates upstream regulators of mTORC1 during pharmacologically induced ER stress [74]. Simultaneously, ER stress appears to suppress the activation of mTORC2 [75]; however, further studies are needed to evaluate whether there is a bidirectional relationship between ER stress and mTORC2 or if ER stress can stimulate mTORC2 in specific contexts. Importantly, activation of both mTOR signaling and the UPR promote cell survival during early signaling stages, but chronic ER stress and sustained mTORC1 activity positively reinforce one another in shifting the cell toward proapoptotic pathways.

Though there are limited studies on the specific mechanisms of mTOR-mediated ER stress in *β*-cells, they are generally consistent with findings from other tissues and cell types. Gene expression analysis of *db/db* islets show associations between ER stress markers and increased ribosomal biogenesis that may be suggestive of increased mTORC1 activity directed by prolonged hyperglycemia [76]. Concurrent with this finding, constitutive mTORC1 activity in *β*-cells through genetic ablation of its upstream regulator TSC2 leads to ER stress *in vivo* that impairs *β*-cell function [22]. Mice in this model have improved glucose homeostasis in early life due to profound expansions in *β*-cell mass but become diabetic due to *β*-cell overwork and cell death. By contrast, inhibiting mTORC1 in human islets treated with lipotoxic and pharmacological ER stressors blunts the UPR and improves *β*-cell survival and GSIS [77]. Similarly, ER distension and proinsulin backlogging induced by lipotoxic conditions in INS-1E rat insulinoma cells can be ameliorated by stimulation of AMPK, which suppresses mTORC1 activity [78]. mTORC1 inhibition is also beneficial for *β*-cell function and glucose homeostasis in Akita mice, a model for ER stress-induced diabetes [79]. In addition to contributing to ER stress under genetic and lipotoxic circumstances, mTORC1 appears to synergize gluco- and lipotoxic conditions in *β*-cells. High glucose is known to exacerbate *β*-cell death caused by lipotoxic ER stress, and this is in part due to mTORC1-dependent upregulation of IRE1 and JNK. This asymmetric enhancement of the UPR specifically amplifies proapoptotic ER stress signals, compounding the effect of lipotoxicity [80]. Taken together, these studies highlight an important role for nutrient-driven mTORC1 overactivation in disruption of ER homeostasis that leads to *β*-cell dysfunction and apoptosis.

### 2.3. The Hexosamine Biosynthetic Pathway, O-GlcNAcylation, and ER Dysfunction

Similar to the case of mTOR, there is a complex interplay of cross-regulation between the UPR and HBP such that induction of one pathway commonly augments the other. HBP enzymes are upregulated by XBP1 in response to ER stress [81], while HBP flux generates metabolic intermediates that initiate ER stress [82]. In particular, high levels of glucosamine directly induce ER stress [83] and cause proteins to accumulate within the ER lumen by impairing N-linked glycosylations [84]. Downstream of the HBP, protein O-GlcNAcylation by OGT is elevated in response to pharmacological induction of ER stress [85] and is cytoprotective against ER stress and CHOP-mediated cell death in a number of cellular contexts [86–88]. Furthermore, suppression of translation by phospho-eIF2*α* depends on OGT-mediated recruitment of proteins to cytoplasmic complexes that stall and degrade mRNA [89]. By contrast, modification of eIF2*α* by OGT allosterically prevents its phosphorylation at Ser51, thereby impeding its downstream induction of CHOP and promoting cell survival [90]. The effect of hyper-O-GlcNAcylation on ER homeostasis in *β*-cells has not yet been investigated, though there are studies highlighting an important role for OGT in *β*-cell ER function. In murine *β*-cells, reducing O-GlcNAcylation through deletion of OGT contributes to ER stress-induced apoptosis that prompts diabetes, while concomitant ablation of CHOP in these mice delays the progression of hyperglycemia [7].

OGT also plays an important role in calcium homeostasis. Fatty acid-stimulated insulin secretion is also disrupted by OGT deletion due to reduced activity of the ER calcium pump SERCA2 [39]. The counterparts of SERCAs are ER calcium release channels, which promote calcium efflux from the ER in response to glucose and stimulation of secretion. The predominant release channel expressed in *β*-cells is IP_3_ receptor 3 (IP_3_R-3) [91]. IP_3_R-3 channel activity is increased by O-GlcNAcylation [92], potentially allowing *β*-cells to fine-tune insulin secretion to nutrient status. Based on the regulation of eIF2*α* and ER calcium transporters by OGT, inappropriate cellular O-GlcNAcylation during T2D may impair the UPR and initiate ER stress, thereby disrupting *β*-cell insulin synthesis, secretion, and cell survival. However, further studies are greatly needed to assess how OGT contributes to ER dysfunction in islets of T2D patients and mouse models.

### 2.4. Defects in Secretory Granules

The ER plays an essential role in insulin biosynthesis, but *β*-cell dysfunction can also stem from defects at the distal end of the secretory pathway in the secretory granules where proinsulin is processed to insulin. Therefore, we will briefly discuss secretory granule physiology and potential roles for mTOR and OGT in the regulation of these organelles. Parameters such as the number, size, shape, and core density of secretory granules are perturbed in *β*-cells of T2D individuals [93] and animal models [94–96] and are signs of dysregulation of insulin granule biogenesis and maturation. Granule maturation requires acidification of the granule lumen, high concentrations of zinc and calcium ions, and activity of proinsulin processing enzymes [97]. Disruption to these factors decreases the secretion of functional insulin. In addition, backlogging of proinsulin within the secretory pathway can stimulate ER stress; this is an additional mechanism by which palmitate induces ER stress [98]. Granule pH is regulated by vacuolar-(v)-ATPase proton pumps located in the secretory granule membrane [99]. Assembly of the v-ATPase complex is coupled to glycolysis [100], suggesting nutrient regulation of secretory granule acidification. Proteomic studies in neurons suggest putative O-GlcNAcylation sites on the v-ATPase complex [101, 102], and loss of OGT causes *β*-cells to display defects in proinsulin-to-insulin processing [103]. These findings may suggest malfunctional granule acidification when OGT is dysregulated, but these studies have not yet been performed in *β*-cells. In terms of secretory granule cation concentrations, Znt8 acts as the major transporter for zinc into *β*-cell secretory granules. Znt8 genetic polymorphisms have been associated with variations in the risk of T2D [104]. This may be due to defects in *β*-cell function, as deletion of Znt8 from *β*-cells reduces the number of insulin granules, the amount of mature granules, and proinsulin-to-insulin processing [105]. Intriguingly, EM imaging of Znt8^—/—^*β*-cells resembles *β*-cells lacking mTOR, which have reduced mRNA expression of Znt8 and lower granularity despite normal proinsulin processing [106]. In terms of proinsulin processing, high levels of palmitate disrupt carboxypeptidase E (CPE), the enzyme that catalyzes the final step in proinsulin-to-insulin conversion within secretory granules [98]. Loss of CPE activity and proinsulin processing is also present in several mouse models of diabetes [107–109] including those due to loss of mTORC1 signaling [110] and deletion of OGT [103]. Altogether, these findings demonstrate significant disruptions to secretory granules during T2D, which can contribute to additional ER stress and impair *β*-cell function. There appear to be links between nutrient-sensing proteins and secretory granule homeostasis, but additional studies in this area are warranted.

## 3. Mitochondrial Dysfunction

While the ER plays an important role in insulin biosynthesis, mitochondria are pivotal organelles in functional insulin secretion. In the classical pathway of insulin secretion that couples secretion to nutrient status, increased availability of substrates for mitochondrial respiration such as glucose and fatty acids increases production of ATP. Elevated cytosolic ATP concentrations then cause closure of K_ATP_ channels in the plasma membrane. Subsequent depolarization of the membrane opens voltage-gated calcium channels and the influx of calcium promotes secretory granule fusion with the plasma membrane [111]. K_ATP_-independent pathways involving cAMP, NADPH, long-chain acyl-CoA derivatives, amino acids, and superoxide have also been described, and these pathways also link mitochondrial metabolism to insulin exocytosis [112, 113]. Importantly, critical roles have been established for dysfunctional *β*-cell mitochondria in the pathology of T2D [114].

Mitochondrial dysfunction is largely characterized by excessive generation of reactive oxygen and nitrogen species (RONS). Chronic oxidative stress is a central mechanism in *β*-cell glucotoxicity, and reactions between cell components and mitochondrial RONS disrupt the metabolic pathways, organelles, and genes required for *β*-cell function [115]. In addition to cell damage, a high oxidative burden activates stress response pathways such as nuclear transcription factor-kappa B (NF-*κ*B) and JNK that impair *β*-cell function and promote *β*-cell death if sustained [116, 117]. *β*-cells are particularly susceptible to oxidative stress as they have been reported to express low levels of antioxidant enzymes [118] and less efficient DNA repair systems compared to other tissues [119].

Glucotoxicity can promote oxidative stress through a number of metabolic pathways in *β*-cells [115], but dysfunctional mitochondria are major drivers of oxidative damage and have been linked to impaired GSIS induced by high-nutrient conditions [120, 121]. Nutrient excess creates a surplus of electron carriers (e.g., NADH and succinate) within the mitochondria. When electron donation to the ETC from electron carriers exceeds the rate of oxidative phosphorylation, electron “leakage” from ETC complexes generates superoxide that can create other RONS and cause oxidative damage. As they are a major site of RONS generation during nutrient excess, mitochondria are poised to incur oxidative damage, a major cause of mitochondrial dysfunction. Intriguingly, inadequate nutrient conditions can also spur oxidative damage *in vitro* in rat islets [122] and INS1 cells [123], suggesting a complex relationship between nutrient status and RONS generation.

In addition to increased RONS production, changes in respiratory function, mitochondrial enzyme expression, mass and volume, morphology, biogenesis, turnover, calcium signaling, and membrane hyperpolarization are also crucial parameters of mitochondrial dysfunction [124]. Mitochondrial RONS cause oxidative damage to cell components outside the mitochondria and also create pernicious feedback loops of RONS production by damaging mtDNA, thereby further impairing proper mitochondrial function [125]. One way that mitochondria can respond to oxidative damage and compensate for mutated mtDNA is through mitochondrial fusion, but this comes at the cost of reduced mitochondrial efficiency that will impair cell function if sustained [125, 126]. By contrast, mitochondrial fission is employed to segregate damaged mtDNA and mitochondrial components, which can then be degraded by mitophagy (selective autophagy of mitochondria) [125]. In addition to fusion, changes in the expression of enzymes for repairing mtDNA (e.g., OGG1) [127] or uncoupling proteins (UCPs) that attenuate RONS production [128, 129] may also signify mitochondrial dysfunction. Finally, inadequate activity of mitochondrial transcription factor A (TFAM) can impair mitochondrial function, as TFAM regulates transcription and replication of mtDNA and is necessary for expression of respiratory complexes and mitochondrial biogenesis [130] as well as stimulus-secretion coupling and *β*-cell survival [131]. These markers are useful tools for assessing mitochondrial dysfunction and many are perturbed in *β*-cells by excessive nutrient exposure.

### 3.1. *β*-Cell Mitochondrial Dysfunction in T2D

Islets from T2D individuals and rodent models are rich with biomarkers for mitochondrial dysfunction that may underlie impairments in *β*-cell function [114]. Markers for oxidative stress are elevated in human T2D islets [132–134] and murine models [135, 136]. Importantly, the onset of diabetes is preceded by elevations in islet superoxide content and markers for oxidative stress that correspond to mitochondrial defects [137] and the degree of GSIS impairment [133]. OGG1 is also upregulated in islet mitochondria of T2D patients, suggestive of a need for mtDNA repair due to high levels of oxidative damage [138]. T2D human [132] and mouse [129] islets also upregulate UCP2 to limit electron backlogging in the ETC and RONS formation. This upregulation reduces *β*-cell oxidative damage but also restricts mitochondrial ATP generation that impairs GSIS [132, 139]. T2D islets also show upregulation of complex I and ATP synthase, which may reflect an insufficient attempt by *β*-cells to offset the energetic costs of controlling RONS production through UCP2 [132].

T2D islets also have pronounced morphological changes indicative of mitochondrial dysfunction and oxidative damage. Mitochondria tend to be enlarged in islets from T2D individuals [132] and several T2D rodent models [140] which reduces oxygen consumption and may signify adaptation to oxidative damage under nutrient stress. At the same time, glucotoxicity induces mitochondrial network fragmentation in human islets [141] and rat models of T2D [142, 143]. Importantly, sustained mitochondrial fusion or fragmentation impairs GSIS, whereas appropriate mitochondrial size and networking are protective against glucolipotoxic cell death [144, 145]. Chronic nutrient excess also reduces TFAM expression and activity in healthy human islets [113] though TFAM defects have not yet been reported in T2D *β*-cells. Altogether, nutrient-stressed *β*-cells demonstrate significant signs of mitochondrial dysfunction that impair functional insulin secretion.

### 3.2. mTOR and Mitochondrial Dysfunction

Mitochondria are extensively regulated by mTOR signaling [146]. The expression of a substantial number of mitochondrial proteins, including TFAM, mitochondrial ribosomal proteins, and several respiratory chain subunits, depends on ribosomal translation involving eukaryotic translation initiation factor 4E (eIF4E). eIF4E is inhibited by eIF4E-binding proteins (4E-BPs), and 4E-BPs are inactivated by mTORC1 [146]. As a result, mTORC1 signaling regulates mitochondrial energy production via regulation of mitochondrial protein translation [147]. Furthermore, mTOR complexes regulate fission, fusion, biogenesis, and mitophagy, thereby controlling mitochondrial mass and turnover as well [146, 147]. Additionally, specific combinations and/or high concentrations of particular amino acids regulate insulin secretion via mitochondrial-driven metabolism-secretion coupling [148]. Increased branched-chain amino acids during T2D and obesity that dysregulate mTORC1 activity may therefore represent another junction for mTORC1-dependent *β*-cell mitochondrial dysfunction [3, 27].

In terms of *β*-cell mitochondrial dynamics affected by mTOR signaling, dysregulated mTORC1 signaling may promote maladaptive changes in mitochondrial function. Though *β*-cell mitochondrial function and biogenesis are improved in the early life of the *β*-cell TSC2 knock-out mouse and contribute to improved insulin secretion [149], constitutive mTORC1 expression eventually causes increased mitochondrial oxidative stress and an accumulation of degenerate mitochondria [150]. This phenotype appears to be largely attributable to defective mitophagy in response to chronic mTORC1 activation. At the same time, the Krebs cycle, ATP production, and GSIS are impaired by rapamycin [151], a compound that inhibits mTOR and from which the kinase derives its name. Deletion of the mTOR kinase from *β*-cells results in defects in mitochondrial membrane potential, reduced mitochondrial activity, and increased oxidative stress, further underscoring the necessity of mTOR signaling in *β*-cell function [12]. Based on these reports that both hyperactivation and loss of mTORC1 signaling lead to mitochondrial dysfunction, mTORC1 activity must be finely regulated in *β*-cells to promote mitochondrial homeostasis. Hence, *β*-cell dysfunction arising from mitochondrial impairments is closely linked to the observation of dysregulated mTORC1 activity during T2D [25].

### 3.3. OGT and Mitochondrial Dysfunction

Mitochondrial function is also finely regulated by O-GlcNAc signaling. Specifically, a spliceform of OGT located within the mitochondria (mOGT) regulates mitochondrial respiration, morphology, and content [152]. A mitochondrial OGA isoform has not been identified, though OGA activity has been demonstrated within mitochondria [153]. Unfortunately, there have not yet been any studies examining the role of O-GlcNAcylation on mitochondrial dysfunction in *β*-cells, though loss of OGT in *β*-cells has been reported to cause abnormal mitochondrial morphology [7]. These morphological changes may be the result of altered O-GlcNAcylation of DRP1 and OPA1, two key proteins that regulate mitochondrial morphology [154, 155], but these mechanisms have yet to be elucidated. *β*-cells lacking OGT also display secretion deficits [7] that may be suggestive of inadequate mitochondrial respiration.

Fortunately, there is extensive research addressing mitochondrial O-GlcNAcylation during diabetes in cardiomyocytes which may provide insights into mitochondrial O-GlcNAcylation in *β*-cells. O-GlcNAc-omic profiling of rat cardiomyocyte mitochondria suggests that at least 88 mitochondrial proteins are O-GlcNAcylated, with respiratory complexes, TCA cycle enzymes, and fatty acid oxidation enzymes comprising the major targets of mOGT [156, 157]. Chronic hyperglycemia produces marked changes in the O-GlcNAc-omic profile of these proteins [157] which can in turn influence their expression and activity [158]. TFAM, a key transcription factor for mitochondrial proteins, is O-GlcNAc modified under high glucose conditions and this modification reduces its mtDNA binding affinity [159]. O-GlcNAcylation also regulates proteins that control mitochondrial fission and increases mitochondrial fragmentation in diabetic rat cardiomyocytes [154, 155]. Increased O-GlcNAcylation during diabetes also reduces the activity of OGG1, an important mtDNA repair enzyme, thereby leading to increased mtDNA damage [160]. Interestingly, it has been suggested that mOGT is localized along the inner mitochondrial membrane under physiological circumstances, but chronic hyperglycemia causes relocalization to the mitochondrial matrix. This altered localization of mOGT corresponds to reduced interactions with complex IV, which may underlie observations of reduced complex IV activity in diabetic mitochondria [153] and would be expected to increase mitochondrial RONS formation. In contrast to this finding, O-GlcNAcylation of complexes I and IV is increased in rats bred to have poor mitochondrial respiration [161], though these factors could be secondary to other changes affecting mitochondrial performance.

Despite these findings, the relationship between changes in O-GlcNAcylation within the mitochondria and mitochondrial function are still not entirely clear. Short-term elevations in mitochondrial O-GlcNAcylation induced through acute pharmacological inhibition of OGA appear to improve mitochondrial function [156, 162], though these observations are not entirely consistent [163]. By contrast, increased O-GlcNAcylation due to chronic hyperglycemia or sustained *in vivo* OGA inhibition generally reduces mitochondrial efficiency [153, 162, 164], though these findings have also been challenged [165]. At the same time, however, reducing mitochondrial O-GlcNAcylation through depletion of OGT or mOGT promotes mitochondrial dysfunction in multiple cellular contexts [152, 162]. Rather than the total amount of mitochondrial O-GlcNAcylation, these studies suggest that long-term deviations from physiological levels of O-GlcNAcylation negatively impact mitochondrial function. Mitochondrial dysfunction may also arise from changes in the rate of O-GlcNAc cycling (i.e., the addition and removal of O-GlcNAc). Tan et al. posit that O-GlcNAc cycling may be more pertinent for protein function than the mere presence or absence O-GlcNAcylation [158]. O-GlcNAc cycling is altered by manipulating the expression or activity of OGT and OGA because these enzymes have linked expression patterns that influence one another, and this may underlie the common observations in the aforementioned studies [158].

Upstream of OGT, the interactions between the HBP and oxidative stress have also been studied extensively [166]. Similar to the relationship between the HBP and ER stress, oxidative damage and the HBP also cross-regulate each other. In general, oxidative stress appears to promote O-GlcNAcylation, but OGT regulation of oxidative damage appears to be more context-specific. For instance, though O-GlcNAcylation can promote the expression of antioxidant enzymes that quench RONS, HBP flux also promotes oxidative damage during glucotoxic conditions. In *β*-cells, GFAT overexpression induces oxidative stress that disturbs *β*-cell function [167]. However, pharmacological inhibition of OGA to increase *β*-cell O-GlcNAcylation does not reproduce this phenotype, suggesting a more important role for HBP flux and glucosamine-induced ER stress rather than changes in protein O-GlcNAcylation [167].

### 3.4. AMPK and Mitochondrial Dysfunction

Though studies of AMPK in *β*-cell organelle homeostasis are limited, there are several that highlight the role of AMPK in *β*-cell mitochondria. There are two isoforms of AMPK's catalytic *α* subunit, and islets from *db/db* mice show repressed expression of the *α*1 isoform [19]. When both *α*1 and *α*2 are deleted from *β*-cells, mitochondria are smaller, fewer in number, and islets reduce transcription of genes for electron transport, oxidative phosphorylation, and mitochondrial biogenesis [19]. However, reducing expression of only the *α*2 isoform appears to have little effect on mitochondrial parameters beyond reducing mRNA expression of UCP2 [168], which may reflect compensation or greater importance of the *α*1 isoform in *β*-cells. Loss of LKB1, an activator of AMPK, produces severe mitochondrial dysfunction and disrupts GSIS [169]. In contrast to reducing AMPK activity, pharmacological stimulation of AMPK in INS-1E cells maintains mitochondrial morphology during lipotoxicity [78] but promotes RONS production and mitochondrial dysfunction in MIN6 cells (a mouse insulinoma cell line) under standard [170] or glucotoxic conditions [171]. Though these findings could also be attributable to differences in the cell lines, they may also suggest a complex role for AMPK and *β*-cell mitochondrial homeostasis that is context-specific.

## 4. Lysosomal Dysfunction

In addition to mitochondria, *β*-cell lysosomes are also critical organelles for regulating insulin secretion and allowing *β*-cells to function efficiently in response to nutrient flux. mTORC1 and AMPK are activated at the lysosomal membrane, making lysosomes hubs for intracellular nutrient-signaling [172]. The lysosomes also regulate the degradation of nascent secretory granules during nutrient deprivation, allowing *β*-cells to limit insulin secretion when it is not needed and providing carbon sources for *β*-cells to metabolize for survival [173]. Additionally, lysosomes are the terminal effectors of the autophagy pathway and function as important sites of macromolecule degradation that allow *β*-cells to adapt to early stages of glucolipotoxicity and remove defective organelles [174]. Despite the importance of this organelle, descriptions of lysosomal dysfunction are not well-established in the literature. In this section, we characterize lysosomal dysfunction as impairments caused by membrane damage, defective biogenesis, and insufficient degradative ability.

Lysosomal damage is primarily characterized by lysosomal membrane permeabilization (LMP). The main driver of LMP is oxidative damage, but lipotoxic conditions that alter lysosomal membrane composition [175], proapoptotic signals, and inflammation can induce LMP as well [176]. Upon LMP, proteolytic hydrolases are released into the cytoplasm. Hydrolases from the cathepsin subclass activate caspases and other proapoptotic proteins to trigger cell death. Cathepsins directly promote mitochondrial outer membrane permeabilization and cytochrome c release. Activated caspases trigger LMP in a positive feedback pathway. LMP can therefore act as both as an upstream initiator and a downstream accelerator for multiple forms of cell death [176, 177]. Cathepsins also promote cell death through mitochondria- and caspase-independent pathways [178, 179]. Mild LMP can prompt lysophagy, i.e., the selective autophagic clearance of damaged lysosomes [180]. Signals from damaged lysosomes also promote inflammatory pathways and inhibit lysosomal biogenesis [181]. The latter outcome prevents cells from producing more lysosomes under stressful conditions that may lead to further LMP but compromises degrative capabilities if sustained.

Lysosomal biogenesis is primarily regulated by transcription factors EB (TFEB) and E3 (TFE3), which stimulate the expression of v-ATPase subunits, acid hydrolases, and trafficking proteins important for lysosomal maturation [182, 183]. TFEB also regulates several genes for autophagy induction and degradation [184]. Nutrient levels [185], ER stress [186], and mitochondrial stress [185] regulate TFEB translocation, suggesting that lysosomal biogenesis is largely a response to metabolic stress that is used to enhance degrative pathways to restore cell homeostasis. However, sustained stress can lead to nuclear exclusion of TFEB, thus limiting lysosomal biogenesis and the ability of cells to manage excessive nutrient influx [185].

Lysosomal dysfunction can also arise when intralumenal enzyme activities are impaired. Deficiencies of key lysosomal enzymes (e.g., hexosaminidases, galactosidases, and cathepsins) cause undigested substrates to accumulate, which contributes to the pathology of lysosomal storage diseases and neurodegenerative disorders [187]. Additionally, inadequate cleavage of procathepsin zymogens disrupts lysosome function, causing lysosomes to swell and impair autophagic flux [188]. The pathogenesis of pancreatitis is in part driven by defective procathepsin processing that disrupts lysosomal activity in this manner [189]. Lysosomal alkalization can also promote lysosomal dysfunction as many lysosomal enzymes require an acidic pH to function. Finally, lysosomal incapacitation can result from the accretion of lipofuscin. Lipofuscin is a crude cross-linked mesh of oxidized macromolecules, metal cations, and sugar residues that accumulates within cells as they age because it cannot be degraded or eliminated [190, 191]. Lipofuscin primarily accumulates within lysosomes, where it impairs hydrolase activity and obstructs autophagic flux [192] while also making lysosomes more vulnerable to LMP from internal oxidative damage [193] due to its propensity to accrue iron and promote ROS generation [194]. Lipofuscin is enriched in aged *β*-cells and may be associated with age-related decline in *β*-cell function [195].

### 4.1. *β*-Cell Lysosomal Dysfunction in T2D

Lysosomal function appears to be perturbed in *β*-cells in response to nutrient excess. Islets from T2D individuals [196, 197] and human islets treated with glucolipotoxic conditions [171] display an accumulation of autophagosomes within their cytosol. These observations may in part be due to lysosomal dysfunction during T2D and glucolipotoxicity, as dysfunctional lysosomes reduce autophagosome clearance, leading to increased prevalence of autophagosomes. In terms of lysosomal damage, LMP is induced in *β*-cells by glucolipotoxicity *in vitro*, likely through mechanisms involving ER stress [188] and enhanced mitochondrial RONS generation [198]. Additionally, inhibiting cathepsin D partially reverses glucolipotoxic cell death in murine islets, highlighting the importance of LMP in nutrient-induced *β*-cell failure [188]. However, it has also been reported that *β*-cells from T2D individuals have reduced transcription of cathepsin B and D [196] and downregulation of lysosome-associated membrane protein 2 (LAMP-2) [199, 200]; these findings may suggest low lysosomal number and reduced TFEB activity. In fact, TFEB translocation to nuclei is reduced in T2D islets [196, 199], and islets from HFD-fed mice show reduced transcriptional expression of TFEB [201]. Furthermore, TFEB and LAMP-2A are decreased in cardiomyocytes of several mouse models of diabetes and obesity, demonstrating more broadly that chronic glucolipotoxicity reduces lysosomal biogenesis through TFEB dysregulation [202]. *In vitro* studies in INS-1 cells reveal that TFEB overexpression exerts a protective effect against glucolipotoxic cell death [201], demonstrating the significance of changes in TFEB expression and activity during T2D. Finally, in terms of disrupted degradation, glucotoxic conditions cause procathepsin accumulation within lysosomes that enhance *β*-cell apoptosis by impairing lysosomal activity and autophagic flux [203]. Similarly, treatment of INS-1 cells with palmitate disrupts lysosome function by reducing lysosomal acidity and cathepsin L activity [204]. Additionally, GSIS is impaired in the *β*-cells of diabetic Goto-Kakizaki rats due to reduced activity of “classical” lysosomal enzymes (e.g., cathepsin D and acid phosphatase) and hyperactivity of *α*-glucosidases associated with malfunctional lysosomal systems [205]. Lysosomal accumulation of lipofuscin, however, does not appear to be more prevalent during obesity and T2D [195], though research in this area is quite limited.

### 4.2. mTOR and Lysosomal Dysfunction

Despite its intimate relationship with the lysosomes, how mTOR may regulate or respond to lysosomal stress has not been thoroughly studied. In myotubules with acid *α*-glucosidase deficiency, enlarged and defective lysosomes demonstrate a reduced ability to release and inactivate mTOR during nutrient deprivation [206]. Similar lysosomal defects in *β*-cells may also hamper mTOR inactivation, though this remains to be studied. In contrast to enzymatic disruption, LMP causes swift and efficient delocalization of mTOR from the surface of the lysosome and initiates robust induction of autophagy at the site of lysosomal damage, thereby limiting the release of lysosomal contents into the cytosol [207]. When mTORC1 is active, however, it prevents lysosomal biogenesis by impairing TFEB translocation and stability [185]. This may support the observation that TFEB is less frequently observed in T2D *β*-cells exposed to chronic nutrient excess with hyperactive mTORC1 [199, 201]. Reduced mTORC1 signaling has been reported to decrease lipofuscin accumulation in several tissues [208, 209], including in the context of T2D [210]; however, it has been reported that the prevalence of lipofuscin in *β*-cells is not increased by T2D or obesity [195]. *β*-cells may have mechanisms for adapting to and limiting lipofuscin accumulation in the context of elevated mTORC1 activity, but additional research will be needed to investigate this matter. AMPK also has important roles in lysosomal membrane integrity [207], biogenesis [211, 212], and hydrolytic ability [213]. However, these roles have yet to be explored in *β*-cells.

### 4.3. OGT and Lysosomal Dysfunction

Hyperglycemia has been used to drive lysosomal dysfunction in many of the studies described above, which would be expected to increase the amount of UDP-GlcNAc-substrate for OGT. However, the direct role of O-GlcNAcylation in lysosome dysfunction has been understudied. OGT recruitment to the cathepsin D promoter and hyper-O-GlcNAcylation of transcriptional corepressors silences gene transcription [214, 215], allowing fine regulation of lysosomal activity by nutrient levels at the transcriptional level. If this same pattern occurs in *β*-cells, the observation of reduced cathepsin D expression in T2D islets [196] may in part result from hyperglycemia-driven O-GlcNAcylation of the transcriptional regulators and machinery at the gene's promoter, though this has not yet been studied. The gene for *β*-galactosidase, another important lysosomal enzyme, may also be subject to O-GlcNAc regulation [216]. Beyond these studies, however, the role of OGT and lysosomal homeostasis is largely unknown.

## 5. Autophagic Dysfunction and Regulation of Organelle Homeostasis

Autophagy is a process characterized by the targeting and delivery of cellular contents and organelles to lysosomes to maintain cellular homeostasis. Autophagy is a critical modulator for *β*-cell organelle homeostasis [217], insulin secretion during fasting conditions [173], and secretory granule turnover [218]. In *β*-cells, autophagy is required to combat glucolipotoxicity, mitigate ER stress, and to maintain healthy pools of organelles in order to protect *β*-cell mass and function during nutrient excess. Genetic deletion of autophagy-related 7 (Atg7), an important protein in this pathway, highlights the importance of functional autophagy as insufficient autophagy impairs *β*-cell proliferation, survival, and insulin secretion [219] and increases *β*-cell vulnerability to lipotoxicity [220, 221].

Several types of autophagy have been described to maintain cell function. Autophagy can be nonspecific or target specific cell components and organelles (e.g., mitophagy to selectively degrade mitochondria), and three major categories of autophagy have been described: macroautophagy, microautophagy, and chaperone-mediated autophagy. An additional form of autophagy relevant to *β*-cells is crinophagy, which involves the direct fusion of secretory granules with lysosomes [222, 223]. Because secretory granule membranes and their associated proteins are recycled rather than degraded, this form of autophagy is an energy-efficient method leveraged by secretory cells to eliminate excess secretory granules [224]. For instance, crinophagy is markedly increased in *β*-cells by genetic deletion of Rab3a, which is an important protein in the secretory pathway [212]. Increasing crinophagy allows *β*-cells to prevent an overaccumulation of insulin granules when levels of insulin biosynthesis are still normal but secretion is impaired. Crinophagy is also responsive to glucose exposure, and the highest rates of induction in murine islets appear to occur at intermediate levels of glucose that promote insulin biosynthesis but do not trigger secretion [223]. Protein kinase D (PKD) is a major regulator of the fate of insulin granules. Loss of PKD signaling leads to constitutive secretory granule degradation, maintains mTORC1 activity, and suppresses macroautophagy [225]. Despite its importance, *β*-cell crinophagy in the context of T2D has not yet been studied, and it is unclear whether defects in this process may be augmented in T2D to contribute to *β*-cell dysfunction. However, there have been a large number of studies examining macroautophagy in the context of T2D, so we will focus our discussion of autophagy on this subtype. Hereafter, “autophagy” in this review refers specifically to the process of macroautophagy.

The mechanisms of macroautophagy have been thoroughly studied and described; briefly, this process involves the sequestration of cytoplasmic components in double-membraned autophagosome vesicles that fuse with lysosomes to deliver their enclosed cargo [226]. Macroautophagy is initiated by the ULK1 complex, which is regulated by mTORC1 and induces Beclin-1 (Becn1) relocation to the ER membrane to nucleate the autophagosome. Further association with several components of the autophagy machinery such as Atg7 and microtubule-associated protein 1A/1B light chain-3 (LC3) is required for the expansion and completion of autophagosome formation. The specificity of autophagy depends on the recruitment of ubiquitylated targets by autophagy receptors such as p62/SQSTM1 that mediate interaction with LC3 proteins embedded in the autophagosome membrane [227]. Once targets are enclosed within the autophagosome, they are trafficked to the lysosomes. The outer membrane of the autophagosome fuses with the lysosomal membrane and subsequently the inner membrane, and enclosed contents are degraded by lysosomal hydrolases.

The two major forms of autophagic dysfunction are impaired autophagic flux and hyperactive induction of autophagy [228]. Impaired flux may arise from insufficient initiation of autophagosome formation or defects in autophagosome-lysosome fusion. The former can result from aberrant suppression of initiators of autophagy by upstream regulators (e.g., nutrient availability or cell stress), whereas the latter may result from changes in fusion proteins, lysosomal alkalization, or insufficient lysosome number. Accumulation of dysfunctional organelles, protein aggregates, or metabolites may signify inadequate autophagic flux. Accumulation of autophagosomes or autophagy proteins (commonly LC3-II, the lipidated form of LC3 associated with autophagosomes) can also indicate impaired autophagy; however, these observations should be combined with additional lines of evidence since they may just as easily indicate hyperactive induction of autophagy [229]. Too much autophagy can disrupt the function of organelles, and dysregulated autophagic signaling can initiate a unique form of programmed cell death [230]. Defects in TFEB activity also contribute to autophagic dysfunction, as TFEB regulates a number of autophagy genes in addition to those for lysosomal biogenesis [184].

### 5.1. Autophagic Dysfunction in *β*-Cells in T2D

A number of studies report perturbed autophagy in the *β*-cells during T2D and gluco/lipotoxicity. *β*-cells from T2D individuals have a higher density of autophagic vacuoles and autophagosomes per unit volume [196] and an accumulation of p62 [231, 232] that may suggest deficits in autophagic flux. Autophagosome and p62 accumulation are also observed in *β*-cells from several T2D mouse models [201, 220, 221, 232]. Furthermore, islets from T2D individuals contain a higher proportion of dead *β*-cells that display signs of autophagy-associated cell death [196]. Treatment of human and rat islets and INS-1E cells with palmitate induces autophagy that confers protection against ER stress-induced *β*-cell death and demonstrates the protective role of autophagy during acute nutrient stress [233–235]. Sustained lipotoxicity, however, appears to impair autophagic flux in islets and INS1 cells [197, 204]. In addition to the lysosomal defects that arise from lipotoxicity, this impairment may be attributable to a shift in autophagic targeting. For instance, in the islets of *ob/ob* mice, total autophagic activity is increased but is skewed toward lipophagy (autophagic targeting of triglycerides and lipid droplets) to combat lipotoxicity, leading to reduced clearance of substrates associated with p62 (i.e., ubiquitylated proteins and organelles) [221]. However, in HFD-induced *β*-cell dysfunction, these impairments of autophagic flux can be attenuated by concurrent intermittent fasting that transiently alleviates nutrient excess [201]. In addition to impairments in autophagic flux, inappropriate activation of autophagy is also detrimental to *β*-cell health and function. Loss of the *β*-cell transcription factor pancreatic duodenal homeobox 1 (Pdx1) increases autophagy and cell death in MIN6 cells which can be delayed with the autophagy inhibitor 3-methyladenine (3-MA) [236]. Similarly, Pdx1 insufficiency *in vivo* causes a loss of *β*-cell mass; however, impairing autophagy through genetic reduction of Becn1 in this same model can preserve *β*-cell mass [236]. In addition to affecting *β*-cell survival, mice with a constitutively-active Becn1 mutant display hyperactive autophagy during HFD feeding and this causes excess degradation of insulin granules and impaired GSIS [237]. While autophagy in human T2D *β*-cells needs to be investigated further, it is clear that sustained nutrient excess disrupts the *β*-cell autophagy system.

Autophagy is an important mechanism for regulating the clearance of defective or stressed organelles. The most studied form of organelle-specific autophagy in *β*-cells has been mitophagy. *β*-cells of diabetic Goto-Kakizaki rats show signs of increased mitophagy due to elevated mitochondrial stress [143, 238]. Additionally, deletion of Atg7 in *β*-cells disrupts clearance of defective mitochondria and causes an accumulation of swollen and deformed mitochondria [191, 219]. Several molecular targets in *β*-cells have been shown to regulate mitophagy. The mitochondrial Rho GTPase Miro1 is critical for *β*-cell mitophagy, and deletion impairs mitochondrial and *β*-cell function [239]. Similarly, overexpression of the regulator of calcineurin 1 (RCAN1), which suppresses autophagy, impairs Miro1-mediated mitophagy in *β*-cells [240]. Furthermore, cytosolic accumulation of p53, via ER and oxidative stress, decreases mitophagy by inhibiting Parkin, an E3 ubiquitin ligase that labels defective mitochondria for degradation [241]. As previously discussed, mitochondrial dysfunction is evident in human islets from T2D donors, increasing the need for mitophagy. However, *β*-cells from islets of T2D humans, diabetic mice, and HFD-fed mice show reductions in Miro1, thereby leading to reduced mitophagy, mitochondrial dysfunction, and impaired insulin secretion [239]. Mitophagy markers in peripheral blood mononucleocytes are elevated in prediabetic individuals, likely as an adaptive mechanism to manage mitochondrial oxidative damage, but progressively decrease with the advancement of T2D. Importantly, these changes correlate with *β*-cell dysfunction, mitochondrial oxidative stress, and increased HbA1C, which may implicate glucotoxicity as a driver of aberrant *β*-cell mitophagy [200]. ER-phagy and lysophagy have not been studied in *β*-cells as much as mitophagy, but still remain important modes for restoring or disrupting homeostasis of their respective organelles. The fact that ER stress and the UPR are major stimuli for autophagy and that autophagy induction helps mitigate ER stress in *β*-cells [79, 242–244] indicates the important role of autophagy in ER homeostasis. Autophagy-deficient *β*-cells lacking Atg7 display ER distension [219] suggestive of luminal protein accumulation and potential ER dysfunction. Autophagy impairments in T2D *β*-cells may therefore disrupt ER turnover and impair the attenuation of ER stress, though this remains to be studied. To our knowledge, there is currently no evidence of *β*-cell lysophagy. However, disrupted lysophagy has been observed in diabetic mouse podocytes and contributes to their dysfunction during hyperglycemia [245]. Future studies of autophagic targeting of these organelles in *β*-cells are greatly needed.

### 5.2. Regulation of *β*-Cell Autophagy by mTOR

In addition to promoting anabolic pathways for cell growth and proliferation, an extensively studied role of mTORC1 is the suppression of autophagy [246]. In the genetic mouse model of constitutive *β*-cell mTORC1 activity through deletion of TSC2, *β*-cells are unable to initiate autophagy in response to ER stress or nutrient deprivation and have impaired autophagosome-lysosome fusion [150]. Similarly, *ex vivo* studies with human islets show sustained mTORC1 activation in response to glucolipotoxicity that suppresses *β*-cell autophagy, leading to dysregulated ER stress and insufficient mitophagy that cause cell death [197]. However, apoptosis caused by lipotoxicity and ER stress can be prevented through rapamycin-induced suppression of mTORC1 and stimulation of autophagy [77, 79]. By contrast, ablation of a key mTORC1 adaptor protein, Raptor, leads to dysregulated *β*-cell autophagy and cell death [110], suggesting important differences between pharmacological and genetic inhibition of mTORC1 in terms of their effect on the regulation of autophagy and *β*-cell survival that may involve timing, duration, and specificity of inhibition. Similarly, stimulation of AMPK in MIN6 cells also promotes autophagy-mediated cell death under standard conditions [247]. However, under lipotoxic conditions, AMPK stimulation is actually protective against *β*-cell death due to autophagy upregulation [247–249]. In general, sustained changes in mTORC1 activity cause dysregulation of autophagy, ultimately disrupting *β*-cell function and survival. While data on AMPK and autophagy is more limited, AMPK appears to be maladaptive when it unnecessarily increases autophagy but can be beneficial for *β*-cells when it is acting as a protective mechanism to nutrient stress.

### 5.3. Regulation of Autophagy by OGT

mTOR signaling is the major regulatory pathway that links the nutrient availability to autophagy, but OGT also acts to fine-tune and amplify autophagic signals [250]. Currently, there are no studies that directly link O-GlcNAcylation to autophagy in *β*-cells. However, regulation of autophagy by O-GlcNAcylation has been studied in other tissues. Increased O-GlcNAcylation of autophagic proteins (e.g., Becn1) is associated with suppression of autophagic flux in cardiomyocytes isolated from *db/db* diabetic mice [251]. O-GlcNAcylation also regulates critical mediators of autophagosome-lysosome fusion in HeLa cell lines and limits their activity [252, 253]. By contrast, O-GlcNAcylation of ULK1 has shown to be an activating posttranslational modification for autophagy initiation in mouse hepatocytes and several human cell lines [254, 255]. Other studies in mouse cortical astrocytes and cancer models show varying degrees of correlation between O-GlcNAcylation and autophagy [256–258]. It is evident that the relationship between O-GlcNAcylation and autophagy is tissue-and context-dependent. Given that *β*-cell O-GlcNAcylation is important for both basal function and adaptation to HFD [7], the relationship between O-GlcNAcylation and autophagy in islets requires further studies.

## 6. Inflammation, Nutrient Sensing, and Organelle Dysfunction in *β*-Cells

In addition to sustained nutrient stress from hyperglycemia and hyperlipidemia, chronic low-grade inflammation has also been well-documented in individuals with T2D and obesity [10]. Longstanding inflammation is characterized by abnormal circulation of proinflammatory cytokines such as tumor necrosis factor *α* (TNF*α*), interleukin-1*β* (IL-1*β*), and interleukin-6 (IL-6). These cytokines contribute to the development of T2D and related complications across multiple organ systems [10]. T2D humans and in animal models, increased levels of cytokines, chemokines, and immune cell infiltration have been observed in islets [259–261]. Chronic islet inflammation has been shown to exacerbate *β*-cell dysfunction and contribute to apoptosis [260–263]. This is in part because *β*-cells both secrete and respond to cytokines [264]. Importantly, chronic hyperglycemia activates inflammatory pathways in *β*-cells [265] and increases cytokine production and subsequent recruitment of inflammatory cells to islets [266, 267]. Several mechanisms of *β*-cell failure discussed in this review including ER stress, oxidative stress, and glucotoxicity can both induce inflammation and result from inflammation induced by excess nutrients [268].

Bidirectionality extends to inflammation and mTOR as well. Inflammation can activate the mTOR pathway through ER stress or TNF-*α*, which may contribute to dysfunctional insulin signaling [269, 270]. mTOR activity can play both protective and detrimental roles in the development of T2D due to tissue-specific differences that may improve or impair cell function. Proinflammatory cytokines activate IKK which promotes activation of mTOR as well as NF-*κ*B, a critical transcription factor that regulates the inflammatory process [271]. In immune cells, mTOR plays important roles in regulating cell metabolism, cytokine production, antigen presentation, macrophage differentiation, and tissue infiltration during the immune response [272]. In the setting of chronic inflammation, inhibition of mTOR with rapamycin reduces islet size and insulin content and increases *β*-cell apoptosis [273]. Beyond this finding, studies in this area are limited, and the interaction of inflammation with the mTOR pathway in *β*-cells is an area in need of future study.

Given the role of O-GlcNAcylation in the cellular response to physiological stresses [274], OGT is also poised to respond and regulate inflammation. In immune cells and in the inflammatory process, O-GlcNAcylation links environmental conditions to intracellular signaling events which can contribute to the development of disease states such as type 1 diabetes and other autoimmune disorders [275, 276]. Multiple studies have shown that there is a complex regulation of the immune system by O-GlcNAcylation that is dependent on the cellular context and nutrient availability [276, 277]. Despite progress in the area, the role of O-GlcNAcylation in islet inflammation remains incompletely understood and warrants further investigation.

### 6.1. Regulation of Inflammation by NF-*κ*B

The NF-*κ*B complex has regulatory roles in innate and adaptative immunity, inflammation, and apoptosis [275, 276]. NF-*κ*B activity has been linked to the pathogenesis of T2D, metabolic syndrome, and related complications [263, 278, 279] by promoting the expression of a number of proinflammatory cytokines including TNF-*α*, IL-1, and IL-6. NF-*κ*B is responsive to high glucose environments, circulating cytokines, and oxidative stress arising from nutrient excess and organelle dysfunction [280]. Chronic NF-*κ*B activity decreases expression of insulin, GLUT-2, and Pdx-1 in *β*-cells [281], and glucolipotoxicity induces NF-*κ*B signaling in *β*-cells that enhances *β*-cell death [282].

mTOR and OGT have been shown to affect multiple components of the NF-*κ*B pathway. mTOR regulates the activation of NF-*κ*B [283] and links NF-*κ*B to hyperglycemia in macrophages [284]. The NF-*κ*B subunits RelA and c-Rel are subject to O-GlcNAcylation, which increases NF-*κ*B transcription activity in the setting of hyperglycemia and in response to TNF-*α* [277, 285, 286]. OGT-mediated O-GlcNAcylation also stimulates NF-*κ*B activity in pancreatic acinar cells during acute pancreatitis [287]. The ongoing exposure to a hyperglycemic state and the activation of NF-*κ*B by mTOR and OGT may therefore contribute to the maintenance of chronic inflammation in islets and the progression of T2D.

### 6.2. Macrophages and IL-1*β*

NF-*κ*B aids in the localization and activation of macrophages at sites of inflammation where they recruit additional immune cells, remove pathogens, and clear cellular debris [278, 288]. Macrophages are a primary source of proinflammatory cytokines in the islet, with IL-1*β* being a key cytokine promoting *β*-cell dysfunction [267]. NF-*κ*B regulates monocyte differentiation into M1 or M2 macrophages, which have pro- and anti-inflammatory phenotypes, respectively [289]. Islets treated with glucolipotoxic conditions demonstrate increased chemokine production [290]. Chronic hyperglycemia and obesity have been observed to promote the development and recruitment of macrophages with a proinflammatory phenotype in other tissues [276, 291]. Activation of the M1 phenotype is favored in the setting of islet inflammation in diabetic mouse models and contributes to *β*-cell dysfunction [292]. Once activated, islet macrophages produce IL-1*β* and TNF-*α*, which act on the *β*-cell to further promote the production of chemokines and additional macrophage recruitment in an autostimulatory manner [293, 294]. Importantly, suppression of M1 macrophage recruitment to islets of *db/db* mice improves glucose tolerance and GSIS [293]. By contrast, M2 macrophages appear to mediate *β*-cell proliferation [295].

Elevated IL-1*β* has been shown to be involved in impaired insulin secretion and insulin resistance [296]. Human islets secrete IL-1*β* in response to chronic hyperglycemia, promoting NF-*κ*B activation, differentiation of macrophages to the M1 phenotype, and contributing to islet inflammation that reduces insulin secretion [297]. There is evidence that hyperglycemia, ER stress, and oxidative stress induce IL-1*β* secretion from *β*-cells and macrophages through activation of the NLRP3 inflammasome [298, 299]. Inflammasome complexes are involved in the processing of IL-1 and IL-18 to their active forms via activation of caspase 1, and upregulation of the NLRP3 inflammasome has been observed in T2D patients [300]. IL-1*β* is also able to stimulate its own expression in an autocrine fashion due to the high expression of the IL-1 receptor on the *β*-cell membrane [301]. HFD-fed rats treated with an IL-1 receptor antagonist show improvements in hyperglycemia, islet insulin biosynthesis, insulin sensitivity, and decreased macrophage islet infiltration [302]. IL-1*β* also links hyperglycemia to *β*-cell apoptosis by increasing *β*-cell expression of the FAS receptor in response to high glucose conditions, which promotes the extrinsic apoptotic pathway when stimulated [297, 303].

### 6.3. Inflammation and *β*-Cell Stress

*β*-cells are sensitive to damage evoked by immune system responses and proinflammatory cytokines elevated during chronic inflammation mediate several modes of organelle dysfunction in *β*-cells [264, 268]. Chronic treatment of *β*-cells with IL-1*β* increases the expression of inducible nitric oxide synthase (iNOS) [304], which is mediated by NF-*κ*B [281], further increasing the expression of proinflammatory genes [304]. Cytokine-induced NO synthesis decreases SERCA2b expression and depletes ER calcium, leading to ER stress [305]. Activation of the UPR promotes further expression of TNF-*α*, IL-1*β*, and IL-6 [306, 307]. One important mediator between ER stress and inflammation in *β*-cells is thioredoxin-interacting protein (TXNIP), which is activated by the IRE1 and PERK branches of the UPR and stimulates IL-1*β* transcription which ultimately promotes apoptosis via NLRP3 activation [308]. Sustained cytokine treatment also promotes ER stress-mediated *β*-cell apoptosis through activation of death protein 5 [309] and upregulation of miRNAs that regulate CHOP [310]. Pharmacological remediation of *β*-cell ER stress has been found to be protective against IL-1*β* induced apoptosis, further highlighting this connection [311]. Increased NO induced by IL-1*β* also reduces mitochondrial ATP synthesis [304], disrupting insulin secretion [312]. Furthermore, cytokine treatment induces mitochondrial network fragmentation and swelling [244]. ER stress-induced by cytokine stimulation of INS-1E cells and rat islets initiates autophagy [244], which may act as a protective mechanism against inflammation-induced *β*-cell dysfunction. Despite increased initiation, cytokines impair autophagic flux by promoting lysosomal alkalization and LMP [244]. Inflammation has broad effects on organelle function, but studies specific to *β*-cells are warranted given the chronic inflammation associated with T2D.

## 7. Concluding Remarks

**Figure 1:**
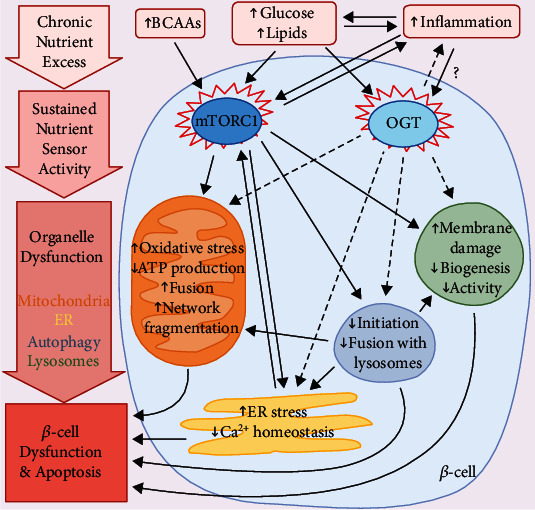
The pathology of *β*-cell dysfunction and failure during chronic nutrient excess: schematic summary of organelle dysfunction driven by dysregulated activity of mTORC1 and OGT. Type 2 diabetes (T2D) and obesity are characterized by chronically elevated hyperglycemia, hyperlipidemia, and elevations in branched-chain amino acids (BCAAs) and inflammation. These conditions activate two key intracellular nutrient sensors in *β*-cells: mechanistic target of rapamycin complex 1 (mTORC1) and O-linked N-acetylglucosamine transferase (OGT). Sustained activity of either of these nutrient sensors leads to comprehensive organelle dysfunction in *β*-cells. mTORC1 activity induces mitochondrial oxidative stress, impairs ATP production, and induces morphological and networking changes that disrupt mitochondrial health and function. In the endoplasmic reticulum (ER), mTORC1 hyperactivity causes ER stress, which can reinforce mTORC1 activity in positive feedback. mTORC1 suppresses autophagosome formation and fusion with lysosomes, which can impact the homeostasis of other organelles such as the mitochondria and ER. mTORC1 hyperactivity also induces lysosomal membrane damage, reduces biogenesis, and impairs the activity of lysosomal enzymes. mTORC1 also has a bidirectional relationship with inflammation that can strengthen these upstream signals. Published studies of increased OGT activity in *β*-cells during T2D have been more limited, but OGT generally follows similar patterns to mTORC1 in terms of its effects on organelle function. As the mitochondria, ER, autophagy, and lysosomes are each critical to the activities within *β*-cells, disruption of mTORC1 and OGT signaling leads to *β*-cell dysfunction and, if sustained, *β*-cell apoptosis. Black arrow: promotes indicated organelle dysfunction; dotted arrow: appears to promote indicated organelle dysfunction in some but not all contexts.

The ER, mitochondria, lysosomes, and autophagosomes are fundamental regulators of *β*-cell function and an increasing number of reports highlight their role in glucolipotoxic and proinflammatory conditions in T2D. Chronic exposure to nutrients and proinflammatory cytokines during T2D promotes organelle dysfunction that contributes to *β*-cell dysfunction and the progression of T2D. Nutrient-sensing pathways are major regulators of organelle homeostasis. In general, acute activation of mTOR and OGT enhances organelle function in response to nutrient elevations. However, dysregulated activity driven by chronic nutrient excess and inflammation during T2D leads to profound changes in the ability of *β*-cell organelles to fulfill their roles (Figure 1). Beyond merely affecting organelle function, mTORC1 and OGT hyperactivity can nudge organelles toward initiating proapoptotic pathways that enhance *β*-cell death during T2D. Inflammation enhances apoptotic signals induced by organelle dysfunction in *β*-cells, and the inflammatory process is regulated by mTOR and OGT. However, our knowledge on the roles of these nutrient sensors is evolving and in need of further research. There are many studies that examine mTORC1 hyperactivity and *β*-cell function and many that demonstrate the role of mTORC1 in ER, mitochondrial, lysosomal, and autophagic function and dysfunction. However, there are relatively few that link mTORC1 hyperstimulation during T2D to organelles within the *β*-cells specifically. There is a similar case for OGT, but this nutrient sensor has been studied even less in *β*-cells than mTOR. Additional studies in these areas are critical for understanding *β*-cell physiology and pathology during T2D. Antidiabetic medications such as GLP1-receptor agonists have been shown to improve *β*-cell function by restoring organelle homeostasis [313]. These molecules can help mitigate ER stress [314–316], improve mitochondrial performance [317, 318], and restore lysosomal function and autophagic flux under glucolipotoxic conditions [319]. However, despite the fact that mTORC1 or OGT broadly regulates many parameters of *β*-cell organelle homeostasis, therapeutics targeting these nutrient sensors in *β*-cells have not been developed. Molecular inhibitors have been identified for mTORC1 [320–322] and OGT [323–325], though many of these putative therapeutics have not been tested in the context of T2D. However, it has been suggested that intermittent dosing with rapamycin can preserve *β*-cell function and glucose homeostasis, which are negatively impacted by sustained rapamycin exposure [326, 327]. This treatment regimen has not been applied to humans, and its specific impacts on *β*-cells need to be further explored, but it may be a promising approach. Advancing our understanding of how nutrient sensors function within *β*-cells will allow better development and assessment of therapies and treatments for *β*-cell dysfunction during T2D and will therefore help mitigate the accelerating burden of this disease on nations and individuals.
